# Development of a Web-Based Monitoring System for Power Tilt-in-Space Wheelchairs: Formative Evaluation

**DOI:** 10.2196/13560

**Published:** 2019-10-26

**Authors:** Charles Campeau-Vallerand, François Michaud, François Routhier, Philippe S Archambault, Dominic Létourneau, Dominique Gélinas-Bronsard, Claudine Auger

**Affiliations:** 1 School of Rehabilitation Faculty of Medicine Université de Montréal Montreal, QC Canada; 2 Centre for Interdisciplinary Research in Rehabilitation of Greater Montreal Montreal, QC Canada; 3 Interdisciplinary Institute for Technological Innovation Department of Electrical Engineering and Computer Engineering Université de Sherbrooke Sherbrooke, QC Canada; 4 Department of Rehabilitation Faculty of Medicine Université Laval Quebec City, QC Canada; 5 Center for Interdisciplinary Research in Rehabilitation and Social Integration Centre intégré universitaire de santé et de services sociaux de la Capitale-Nationale Quebec City, QC Canada; 6 School of Physical and Occupational Therapy Faculty of Medicine McGill University Montreal, QC Canada

**Keywords:** wheelchairs, eHealth, health behavior, pressure ulcers, self-help devices, remote sensing technology, technology assessment

## Abstract

**Background:**

In order to prevent pressure ulcers, wheelchair users are advised to regularly change position to redistribute or eliminate pressure between the buttocks region and the seat of the wheelchair. A power tilt-in-space wheelchair (allowing simultaneous pivoting of the seat and the backrest of the wheelchair toward the back or front) meets many clinical purposes, including pressure management, increased postural control, and pain management. However, there is a significant gap between the use of tilt as recommended by clinicians and its actual usage. A Web-based electronic health (eHealth) intervention, including a goal setting, monitoring, reminder, and feedback system of the use of power tilt-in-space wheelchairs was developed. The intervention incorporates behavior change principles to promote optimal use of tilt and to improve clinical postprocurement follow-up.

**Objective:**

This study aimed to conduct a formative evaluation of the intervention prototype to pinpoint the functionalities needed by end users, namely, power wheelchair users and clinicians.

**Methods:**

On the basis of an evaluation framework for Web-based eHealth interventions, semistructured interviews were conducted with power wheelchair users and clinicians. A content analysis was performed with a mix of emerging and a priori concepts.

**Results:**

A total of 5 users of power tilt-in-space wheelchairs and 5 clinicians who had experience in the field of mobility aids aged 23 to 55 years were recruited. Participants found the Web interface and the physical components easy to use. They also appreciated the reminder feature that encourages the use of the tilt-in-space and the customization of performance goals. Participants requested improvements to the visual design and learnability of the Web interface, the customization of reminders, feedback about specific tilt parameters, and the bidirectionality of the interaction between the user and the clinician. They thought the current version of the intervention prototype could promote optimal use of the tilt and improve clinical postprocurement follow-up.

**Conclusions:**

On the basis of the needs identified by power wheelchair users and clinicians regarding the prototype of a power tilt-in-space wheelchair monitoring system, 3 main directions were defined for future development of the intervention. Further research with new wheelchair users, manual tilt-in-space wheelchairs, various age groups, and family caregivers is recommended to continue the formative evaluation of the prototype.

## Introduction

### Background

Globally, about 65 million people need a wheelchair [1]. In North America, an estimated 15% of wheelchair users living in a community use a power wheelchair [2,3]. Pressure ulcers represent a major problem for power wheelchair users [4]. The loss of mobility and lack of sensitivity are important risk factors in the formation of pressure ulcers [5]. For example, over 50% of Americans with spinal cord injuries develop at least one pressure ulcer during their lifetime [6]. The risk of developing pressure ulcers also affects other wheelchair users with central neurological conditions (eg, multiple sclerosis and cerebral palsy) [7,8] and elderly people who experience fragility associated with a major loss of mobility [9,10]. In addition to causing pain and infections and increasing mortality risk, a pressure ulcer may require hospitalization of 6 to 14 days [11,12] along with extended bedrest. Consequently, the presence of a pressure ulcer may not only limit an individual’s capacity to participate in significant activities [13] but may also detract from their quality of life [14,15]. In addition, pressure ulcers have a major financial impact on the health care system: the estimated cost of their treatment ranges from Can $2000 to Can $20,000, depending on their severity [16].

Scientific studies [17,18] and the best-known practice guides [19,20] recommend that users increase blood flow to the buttocks region by regularly changing position to redistribute or eliminate pressure between the buttocks region and the seat of the wheelchair, while avoiding sliding on the seat surface. To do so, depending on the individual’s capacities, several strategies can be used to reduce pressure on the buttocks region (eg, pushing up, leaning forward and sideways, and positioning oneself on the back wheels) [21]. However, some users are unable to complete these maneuvers and, therefore, need to activate power tilt on their wheelchair to compensate for their inabilities. Power tilt allows simultaneous pivoting of the seat and the backrest of the wheelchair toward the back (or front). The constant seat-backrest angle keeps the user at the back of the seat, preventing friction and sliding during a change of position. Depending on the angle of the tilt, the pressure on the seat decreases by 11% to 55% [22-24]. To optimize the benefits of power tilt in reducing pressure, it is recommended that wheelchair users tilt every 30 min, for at least 1 to 2 min [19] at a minimum angle of 30 [22,25].

Therefore, the use of power tilt is an effective means of changing the pressure distribution between the buttocks region and the seat, as needed, because it redistributes pressure largely to the backrest of the wheelchair [26]. In fact, the use of power tilt provides benefits beyond prevention and treatment of pressure ulcers. On the basis of a literature review followed by focus groups, Lacoste et al [27] identified the main reasons that power wheelchair users use tilt daily: (1) comfort and pain, (2) rest and relaxation, (3) posture, (4) functional independence, and (5) physiological functions. The participants claimed that they used tilt for comfort purposes (95%), rest (92%), relaxation (70%), or pain reduction (77%). Only 30% of the participants reported tilting during the day to prevent or treat pressure ulcers, and 20% of them used tilt to avoid sliding on the seat of their wheelchair. They also reported that they tilted at small (0°-15°) and medium (16°-30°) angles much more often than at large angles (31°-45°). This observation has been corroborated by several other studies [18,28-30] that all reached the same conclusion: there is an important gap between the usage recommended by clinicians and the actual use of power tilt.

Personalized instruction in the proper daily use of tilt is indeed part of the care continuum of power wheelchair users. However, recently documented clinical practices demonstrate that little or no time is dedicated to training sessions and practice using various wheelchair components [31-36]. Furthermore, given that the conceptualization of reasons for the use of power tilt is complex and differs greatly between clinicians and users [30], it may be difficult for both parties to reach a common understanding of the recommended use of tilt during power wheelchair procurement. Under these circumstances, it is understandable that the lack of postprocurement follow-up of the use of mobility aids is one of the main concerns of wheelchair users [37].

To date, several studies have examined monitoring technologies to gather objective data regarding the use of mobility aids. The scoping review by Routhier et al [38] pertaining to the use of monitoring technologies by power wheelchair users found that activities associated with the prevention and treatment of pressure ulcers are the most frequent research topic. Among the 43 studies compiled, only 1 proposed an intervention involving interaction between clinicians and users where clinicians could objectively monitor the daily use of power tilt and other wheelchair components (reclining backrest, elevating leg rest, and seat) [39]. Recently described by Wu et al [40], this intervention, which is offered in the form of a mobile app, is intended to prompt users to adopt the repositioning behaviors recommended by their clinicians by issuing reminders and personalized alerts. Nonetheless, although this intervention is on the market, only a very small number of users and clinicians can benefit from it because it is compatible with power wheelchairs from only 1 manufacturer. A portable monitoring system that could be installed on various models of power wheelchairs would reach a wider range of users.

### Objectives

Consequently, our research team has developed a Web-based electronic health (eHealth) intervention that integrates technology and professional advice. The prototype includes a monitoring system that can be installed without complex manipulation or permanent modification on all models of power wheelchairs. The data gathered are transmitted to the user and to the attending clinician via a Web interface. This intervention aims to favor optimal use of tilt among users of any power wheelchair model and to improve the postprocurement monitoring offered by clinicians. Our study’s objective was to perform a formative evaluation of our monitoring system prototype to clarify the functionalities needed by end users (power wheelchair users and clinicians) and thus increase the likelihood that healthy behaviors targeted by the intervention are adopted. Formative evaluation of a system by end users is typically performed when a product is in the early stage of its development to identify and solve problems that influence the end user’s experience [41,42].

## Methods

### Prototype Description

The proposed intervention was developed by a multidisciplinary team of researchers, clinicians, students, and business partners working in the fields of rehabilitation and electrical and computer engineering. The Behavioral Intervention Technology (BIT) model [43] illustrates the components of the intervention (Table 1). Already commonly used in the eHealth domain [44-46], the BIT model has the advantage of reconciling principles issuing from behavioral change theories with different concepts in electrical and computer engineering. This model describes 2 conceptual components and 3 technical components to consider during the development of eHealth interventions, namely, the aims of the intervention, behavioral change strategies, elements, characteristics, and workflow.

The literature was reviewed to compile the clinical goals associated with the use of tilt. In addition, identification of the needs and priorities of stakeholders [47] enabled us to select behavioral change strategies (eg, feedback on performance), elements (eg, transmission of data concerning the use of tilt), and characteristics (eg, graphics and text results) to include (Table 1). The reference framework proposed by Webb et al [48] on effective behavioral change strategies used in eHealth interventions has also guided the choice of behavioral change strategies.

**Figure 1:**
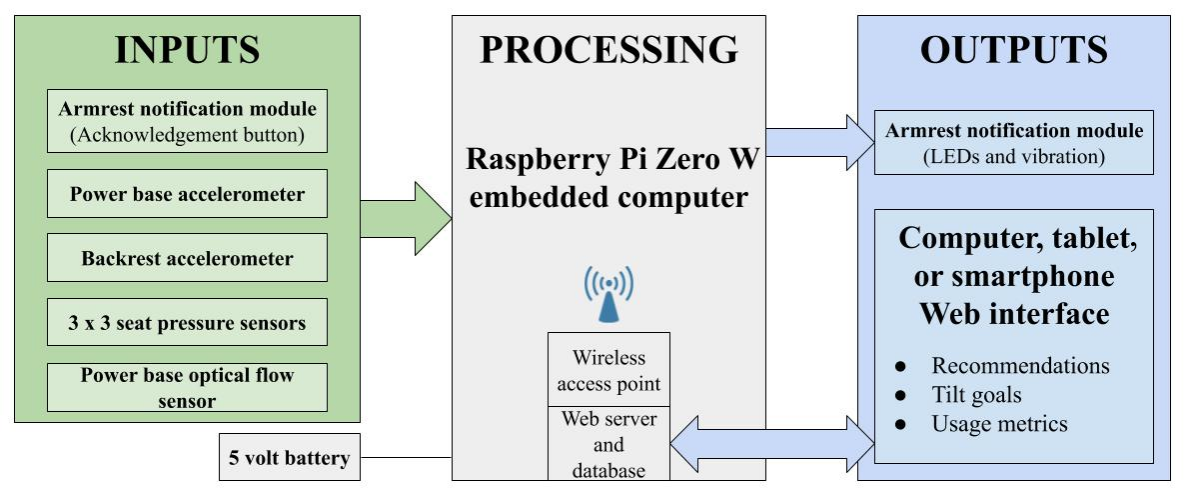
Embedded computing system architecture performing data acquisition, storage, and processing on a power tilt-in-space wheelchair.

Figure 1 presents the prototype of the developed monitoring system. The system includes 2 accelerometers (InvenSense MPU-6050) installed at the power base and backrest of the wheelchair. Each of them measures the tilt angle relative to the direction of gravity, then the difference of both angles provides the effective tilt angle, independent from the surface unevenness. A matrix of 3 × 3 sensors (Interlink Electronics FSR 400) measures pressure on the seat to activate the monitoring system when someone sits on the wheelchair, and the information is used to calculate time spent in the wheelchair. An optical flow sensor (PMW3901) detects movement of the wheelchair to send alerts when stationary only. An embedded computing system (Raspberry Pi Zero W) calculates the time seated in the wheelchair and the tilt time. The computer analyzes the results, archives them in a database, and displays them on a Web interface accessible by a local wireless network. The system also includes a notification module equipped with indicator lights emitting diodes and a vibration motor that serves as a tilt reminder. No personal data are stored in the embedded computing system as it is linked to an external secured server for data management and security.

**Table 1:** Monitoring system of the use of the power tilt wheelchair according to the Behavioral Intervention Technology model.

Conceptual and technical components of the Behavioral Intervention Technology model	Power tilt-in-space monitoring system
Aims of the intervention (conceptual “Why”)	Favor optimal use of power tilt Allow clinicians to offer users more effective postprocurement follow-up of power tilt
Behavioral change strategies (conceptual “How”)	Provide information on the outcomes in general: inform users of tilt parameters (frequency, angle, and duration of tilt) associated with recommended tilt goals Provide information on the outcomes for individuals: inform users about reasons linked to recommended tilt goals Action planning: allow users to create their own personal tilt goals Reinforcing effort toward behavior: recognize users’ efforts to attain recommended and personal goals Provide rewards for behavior: congratulate users on attainment of goals Prompts/cues: issue tilt reminder when necessary Provide feedback on performance: transmit results on daily and monthly use of tilt according to recommended and personal goals
Elements (technical “What”)	Collection, analysis, and passive transmission of data regarding the use of tilt to the user and clinician Reminder (indicator lights and vibration motor) aligned with tilt parameters of personal goals Data entry field
Characteristics (technical “How”)	Medium: text, images, and graphics Complexity: tasks are easy to perform and have simple instructions Aesthetics: simple and discreet
Workflow (technical “When”)	Automatic transmission of results on the use of tilt at specific intervals (eg, at the end of each day or start of each month)

**Figure 2:**
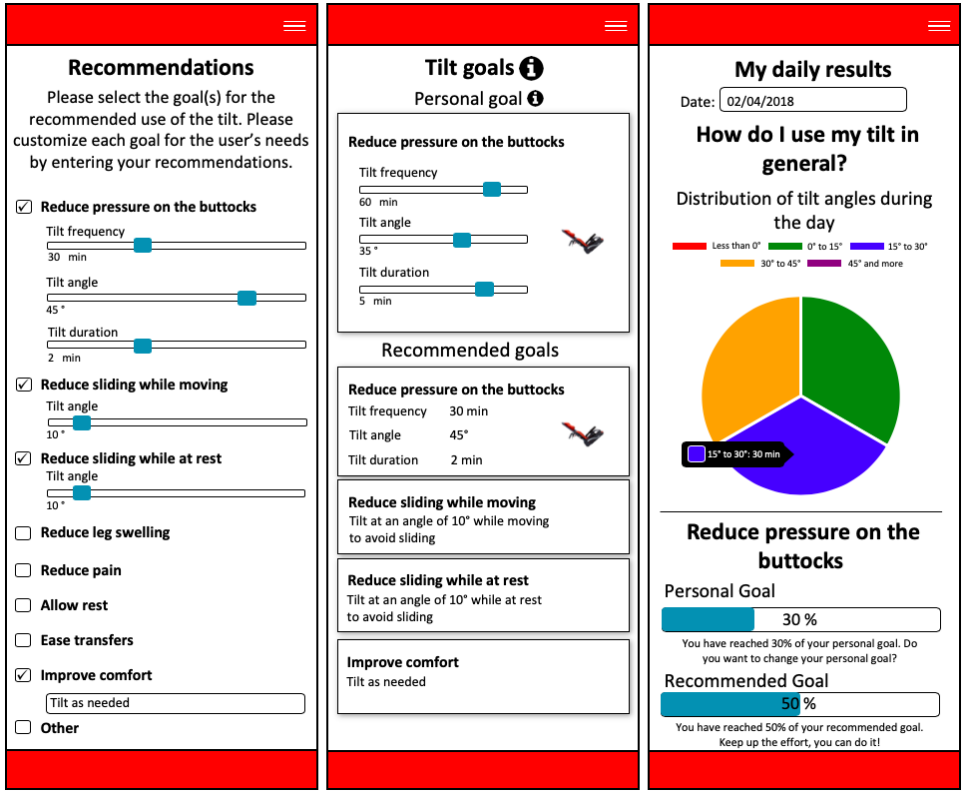
Screenshots of main pages of the Web interface of the power tilt usage monitoring system.

As shown in Figure 2, the monitoring system’s Web interface is optimized for computers, tablets, and smartphones. The Web interface includes separate pages to input recommendations and displays tilt goals and feedback on the use of tilt. On the recommendation page, attending clinicians can specify recommendations from among the proposed tilt goals: (1) prevention and treatment of pressure ulcers, (2) postural control at rest, (3) postural control during movements, (4) edema, (5) pain management, (6) comfort, (7) transfers, and (8) rest. Users can also create personalized tilt goals to add to the list of recommendations. Once the recommendations are saved, they are automatically available to users in the form of recommended tilt goals. These goals are configured to provide information on the positive outcomes of the use of tilt. In addition, at all times, users can personalize their own performance targets and tilt parameters (frequency, angle, and duration of tilt) associated with the goal of prevention and treatment of pressure ulcers. The term *personal goals* refers to new targets set by the user, as opposed to the *recommended goals*, initially set by the clinician. The Web interface also included a section where users and attending clinicians can view daily and monthly data on tilt usage in real time. These data are displayed in the form of graphics and text results that show the user’s performance relative to the recommended and personal tilt goals. A message encouraging users to keep up with their efforts or to try to attain their tilt goals is also displayed. Another element intended to motivate users to use tilt, specifically to prevent and treat pressure ulcers, is the tilt reminder (Multimedia Appendix 1). This reminder is activated each time the user sits in the wheelchair for a period longer than the tilt frequency specified in the user’s personal goal. In addition, indicator lights change color when users reach or exceed the angle and duration of tilt specified in the personal goal to inform users that they have achieved the desired behavior.

### Recruitment

A formative evaluation was performed with 5 users of power tilt-in-space wheelchairs and 5 clinicians who had experience in the field of mobility aids. To ensure that they could easily navigate a Web interface, all participants had to have basic knowledge of how to use a computer, tablet, or smartphone. Users had to be at least 18 years old and use a power tilt-in-space wheelchair as their main mobility aid. There were no exclusion criteria for the 2 groups of participants.

The project received ethical approval from the institutional review board of the Centre for Interdisciplinary Research in Rehabilitation of Greater Montreal (CRIR-1090-0715). The coordinators of clinical research at 3 rehabilitation centers in the Greater Montreal Area (Canada) identified all potential participants. Participants provided written consent to participate in the study.

### Interview Guide Development

To ensure that all concepts that influence the quality of the Web-based intervention were developed and the users’ experience were covered, the conceptual framework by Baumel et al [49] was used to build the interview guide. Accordingly, the following concepts were examined: usability, visual design, content, user engagement, therapeutic persuasiveness, therapeutic alliance, and general subjective evaluation. The interview consisted of 2 parts. First, after having briefly described the prototype and the intervention goals, realistic task-based scenarios were presented to the participants. Specifically, power wheelchair users (1) viewed photos of the physical components of the system installed on a power wheelchair, (2) consulted tilt goals and set a personal goal according to their preferences in the Web interface, (3) viewed a tilt reminder on video, and (4) consulted and interpreted results linked to daily and monthly use of tilt directly in the Web interface. Clinicians viewed the same scenarios, with the addition of a fifth scenario on the entry of recommendations regarding the use of tilt. During the scenarios, participants were asked to think aloud, a process that encouraged participants interacting with a product to verbalize their thoughts, reactions, and emotions, which provided an insight into their experience as a user [41]. Participants were asked open-ended questions after each scenario. The second part of the interview contained questions concerning the prototype in general. A preliminary version of the interview guide was tested with a family caregiver and clinician, both familiar with the mobility aids domain. The comments served to improve and refine the guide (contact CA to obtain a copy).

### Data Collection Procedure

Each of the participants was met individually for a session that lasted about 1.5 hours, which was recorded in a digital audio format. The participants’ sociodemographic data, including their level of perceived experience with technologies and the internet, their reasons for using tilt, and their history of pressure ulcers, were also obtained. Observation notes (eg, participants’ nonverbal reactions) were taken throughout the session.

### Data Processing and Analysis

Interview transcriptions were subject to content analysis with a mixed coding approach. The coding guide was based on the key concepts by Baumel et al [49] (main themes and subthemes), and the emerging themes were linked to these concepts until a final coding guide was developed. Coding was done with QDA Miner v5.0 (Provalis Research) software by the first author (CCV), and a second author (DGB) coded transcriptions independently. Divergence in coding was discussed with the last author (CA) to reach consensus.

## Results

### Study Participants

Participants’ characteristics are presented in Tables 2 and 3. Power wheelchair users’ ages ranged from 23 to 49 years, and clinicians’ ages ranged from 34 to 55 years. Each of the 2 groups of participants comprised 3 men and 2 women. All power wheelchair users had a neurological condition, and 3 of them had pressure ulcers in their buttocks region. They lived in the community and used their device daily as their main mobility aid for at least 8 years. Regarding their current usage of the tilt, they all reported using it for either comfort or to prevent and treat pressure ulcers. Moreover, 3 out of 5 users also mentioned that they used tilt to avoid sliding on their wheelchair, and 1 person mentioned easing transfers. The 5 clinicians interviewed were all occupational therapists. One of them worked in a neuromuscular disease program and the other 4 worked in technical aid programs and services. Perceived experience with technologies and the internet varied within the sample.

**Table 2:** Sociodemographic characteristics of power wheelchair users (N=5).

Sociodemographic characteristics		Value
Age (years), range		23-49
**Gender, n**		
	Male	2
	Female	3
**Principal diagnosis, n**		
	Cerebral palsy	3
	Quadriplegia	2
**Occupation, n**		
	Employed	2
	Unemployed	2
	Student	1
**Level of perceived experience with** **technology and the internet, n**		
	Inexperienced	1
	Somewhat experienced	1
	Very experienced	3

**Table 3:** Sociodemographic characteristics of clinicians (N=5).

Sociodemographic characteristics		Value
Age (years), range		34-55
**Gender, n**		
	Male	2
	Female	3
**Level of perceived experience with** **technology and the internet, n**		
	Inexperienced	1
	Somewhat experienced	3
	Very experienced	1

### Evaluation Outcomes

The 7 main themes of the conceptual framework by Baumel et al [49] captured the viewpoints of users and clinicians. We identified only 4 emerging subthemes, all of which represented the clinical perspective, and they were regrouped under the therapeutic persuasiveness and the general subjective evaluation concepts. Table 4 summarizes the participant’s comments regarding the Web-based monitoring system. These results are presented in detail in the following paragraphs.

**Table 4:** Main participants’ comments about the power tilt usage monitoring system.

Baumel concepts	Users (n=5)	Clinicians (n=5)	Interview results
Usability (ease of use and ease of learning)	4	5	Physical components are convenient for daily use of the wheelchair
	4	5	Web interface is easy to use
	5	5	Some interactive functions of the Web interface are not intuitive
Visual design (appearance and visual quality)	4	4	System looks discreet on the wheelchair
	3	1	Web interface could have more colors
	4	2	Web interface could have larger fonts
Content (content provided or learned during the use of the Web intervention)	5	5	Web tilt goals are well presented to the user
User engagement (extent that the Web intervention employs strategies to attract and encourage its adoption)	3	5	Personal tilt goal for the prevention and treatment of pressure ulcers is appreciated
	4	5	Reminder settings are not appropriate in certain contexts
Therapeutic persuasiveness (extent to which the Web intervention encourages users to make positive behavior change)	5	—^a^	Users felt that the tilt reminder would encourage them to tilt more often
	5	5	Color of the indicator lights according to the angle and the duration of the tilt is appreciated
	5	5	Feedback on the goal associated with the prevention and treatment of pressure ulcers should not take the form of a global analysis
Therapeutic alliance (ability of the intervention to create an alliance with the user to bring about positive change)	4	4	Web interface is missing a space where users can share their experience regarding the use of tilt with their clinicians
General subjective evaluation (potential anticipated benefit of the intervention for the target audience and to the possible usage contexts)	5	5	Participants felt that the goal of the Web intervention was met by the current system
	—	5	Clinicians considered that the Web intervention would improve postprocurement follow-up of tilt use

^a^Not applicable.

#### Usability

Regarding ease of use of the physical components of the system, nearly all participants mentioned that the current configuration of components allowed adequate daily use of the wheelchair. However, they also mentioned several aspects that should be considered during the configuration and installation of components (eg, minimize the overall width of the wheelchair and preserve the possibility to hang personal effects on the backrest). Regarding the Web interface, most users claimed that the entire Web interface was easy to use and navigate. Similarly, all clinicians reported that it was easy to enter the recommendations within a reasonable time. Regarding learnability, all users and clinicians had difficulties with exploring some of the interactive functions of the Web interface at some point, particularly during consultation of their tilt goals and their results. The participants attributed these difficulties to the unintuitive aspect of the functions in question. Almost all the participants (users: n=5 and clinicians: n=4) thought that a training session that included a demonstration would be necessary to learn how to use the system.

#### Visual Design

Almost all participants described the system as discreet when installed on the wheelchair owing to its small size and the black color of its physical components. All the participants appreciated the general structure of the Web interface. However, some participants would have liked to see more colors, particularly during the consultation of the tilt goals, and others would have preferred a larger font.

#### Content

Overall, 4 clinicians found that the reasons for the use of tilt that were displayed in the recommendation entry screen corresponded to those they would normally recommend, whereas 1 clinician mentioned that his practice was restricted to a few on the list. All the participants found that the tilt goals available to users online were presented clearly and appropriately. All the participants also appreciated the clarity of the content of the daily and monthly results of the different tilt goals, except for those associated with prevention and treatment of pressure ulcers. They would have liked the content of the results related to this goal to include more explanatory information such as a written summary of the graphics.

#### User Engagement

All the clinicians appreciated the ability to personalize the recommended tilt goals according to the users’ needs such as the choice of frequency, angle, and duration of tilt and personalized text entry. Most of the participants appreciated that the system let users set their own personal tilt goal in addition to the goal recommended by the clinician for the prevention and treatment of pressure ulcers. For example, 1 user said:

It’s good that you can set a personal goal for yourself because sometimes the occupational therapist may recommend something you are not really used to, but with your personal goal you can calmly go about reaching the recommended objective by increasing your personal goal each day.

User-04

Furthermore, 2 users who were less interested in adopting a personal goal mentioned that they would not set a personal goal at the start of the intervention because they preferred to rely solely on the objective recommended by the clinician.

All the participants appreciated being able to put the tilt reminder in sleep mode at any time. However, almost all of them found the indicator lights and vibration motor of the reminder irritating, too loud, or quite inappropriate in some contexts (eg, at school, work, or the movies). Consequently, they would have liked to be able to personalize the reminder settings according to their preferences (eg, deactivate the vibration motor of the reminder).

#### Therapeutic Persuasiveness

All users thought that the tilt reminder would encourage them to tilt more often. One clinician (Clinician-01) described the reminder as a *mini coach* in charge of motivating users to achieve their tilt goals. In addition, all the users and clinicians who were interviewed appreciated the fact that the indicator lights installed on the reminder box changed color when the user reached or exceeded the angle and duration of tilt set in their personal goal. For example, 1 clinician (Clinician-02) claimed that the synchronization of the indicator lights with the tilt parameters made the parameters much more concrete for users and consequently easier to follow. However, most users (n=4) would have liked to receive tilt reminders not only related to the goal of prevention and treatment of pressure ulcers but also concerning other tilt goals proposed by the intervention (eg, reduce pain and improve postural control when moving) because the degree of attention that these goals require varies greatly during the day. Concerning the pertinence of results regarding the daily and monthly use of tilt, most users (n=3) mentioned that feedback available on the Web interface represented an additional source of motivation to help them achieve their objectives. The 2 users who did not share this view stated that they would not be inclined to view their progress online, but they would rely instead on the tilt reminder as the single source of motivation to achieve their goals. Finally, all users said they would prefer that the feedback on the goal associated with prevention and treatment of pressure ulcers take the form of an individual analysis of each of the tilt parameters rather than a global analysis. For example, 1 user claimed:

In my feedback [on the goal associated with prevention and treatment of pressure ulcers], I would like to be able to isolate information regarding the frequency, angle and duration of tilt so that I could see where I need to improve more easily. This way I could know if, for example, I have to work more on tilt at a larger angle or if instead I should focus my efforts on tilting at a higher frequency.

User-01

#### Therapeutic Alliance

The vast majority of clinicians (n=4) found that the formulation of personal goals by users had a positive influence on their recommendations regarding the use of tilt. According to 1 clinician who shared this view:

[The personal goal] helps me better understand how I as a clinician can give better recommendations because if users find they are obtaining more benefits with their personal goals, this means that my recommendation was not totally adapted to their needs.

Clinician-01

Consequently, these same clinicians believed that setting personal goals favors the creation of dialog between the 2 parties and ultimately of a compromise between what the clinician recommends and what the user is willing to do. Finally, almost all the users would have liked to see a space on their Web interface where they could share their daily and monthly experience with the use of tilt with clinicians. In fact, 4 of the 5 clinicians interviewed confirmed that they would have liked to have access to this form of user feedback because they viewed it as an opportunity to support their clients as they strive to achieve the desired behavior.

#### General Subjective Evaluation

All the participants confirmed that the intervention proposed would favor optimal use of tilt by users. In addition, all the users claimed that they would agree to use the system if it was available. All the clinicians also believed that this monitoring system would let them offer users more effective postprocurement follow-up of tilt use. For instance, 1 clinician mentioned:

I find it interesting that this type of system could offer us information on the use of tilt because when users leave the rehabilitation center, we don’t know what they’re doing with their tilt. When we meet them only every so often, without being in bad faith, they report what they feel is pertinent. No matter how many questions we ask, we will never get as much information as the system can provide.

Clinician-04

However, similar to the users, the clinicians (n=5) would have preferred that feedback on the goal associated with the prevention and treatment of pressure ulcers take the form of an individual analysis of each of the tilt parameters instead of a global analysis. Regarding the feasibility of the intervention in health care institutions, most of the clinicians (n=4) claimed that they would recommend this intervention at the beginning of care to present specific problems such as the appearance of pressure ulcers. In addition, aside from a client at risk or dealing with pressure ulcers, several clinicians (n=3) also found that such a system would be particularly beneficial for users with a degenerative neurological condition, particularly because of the many reasons obliging them to use tilt daily. Finally, 3 clinicians emphasized that the use of this system should not be limited to rehabilitation centers and that it should also be implemented in community health care centers because they too have a role to play in postprocurement follow-up of power tilt.

## Discussion

### Principal Findings

The objective of this study was to perform a formative evaluation of a prototype of a system to monitor tilt use in power wheelchairs. The main results suggested that the physical components and the Web interface were easy to manipulate and use daily. Participants appreciated the tilt reminder and the ability to set their own performance goals. In addition, all the participants expressed an intention to adopt the intervention, and all of them claimed that the current prototype would favor optimal use of tilt by wheelchair users. This confirmation corroborates the findings of other studies regarding the potential benefits of a tilt usage monitoring system [18,40]. The clinicians interviewed also believed that the intervention developed would make postprocurement follow-up of power tilt more effective during different stages of care (preventive or curative), with varied clients and in various practice settings.

Participants’ positive evaluation of the personal goal is certainly one of the most original findings of our study for 3 main reasons. First, users’ comments suggest that the personal goal could serve as an action plan and consequently mediate the gradual attainment of goals recommended by clinicians. Second, the users’ opportunity to create an action plan also guarantees that they can control the use of tilt. This aspect is important because it has been established that for a power tilt usage monitoring system to be adopted by users, they must not feel forced to comply with the recommendations given [50]. Third, the personal goal helps clinicians determine whether their recommendations are truly adapted to the variability of users’ daily occupations. In other words, the personal goal is the representation of what the user is willing to do regarding the use of tilt. This notion of variability of daily occupations, unique to each individual, is important for clinicians because it predicts the real use of power tilt [8,18,29]. Thus, clinicians can better judge whether they need to adjust the recommendations to correspond with the user’s daily routine.

Concerning the tilt reminder, all the users mentioned that it would encourage them to tilt more often. This claim is coherent with a study of the effect of an audio reminder on repositioning behaviors in wheelchairs linked to the prevention and treatment of pressure ulcers [51]. In addition, all the participants appreciated the fact that the indicator lights installed on the reminder box changed color when their tilt reached or exceeded the angle and duration specified in their personal goal. Therefore, this function meets a common need for all participants because research has demonstrated that users and clinicians alike find it difficult to associate the value of an angle with an exact position of tilt without any cues [30].

This formative evaluation highlighted 3 main orientations for improving the future development of monitoring systems for power tilt-in-space wheelchairs. One important area of improvement will be to personalize the reminder settings (indicator lights and vibration motor) according to the context (eg, at school, work, and the movies). This is consistent with the study by Liu [52], which found that preferences in the choice of tilt reminder settings vary depending on the users’ context. Another important change is to ensure that the feedback on the goal associated with prevention and treatment of pressure ulcers takes the form of an individual analysis of each of the tilt parameters rather than a global analysis. The initial prototype presented a combined result of 3 tilt parameters (frequency, angle, and duration of tilt) because this combination predicts greater effectiveness at reducing pressure on the seat [17]. The advantage of offering a global analysis of the attainment of this goal is that participants know the proportion of tilts done according to the 3 parameters of the personal and recommended goals. However, in a context of training and follow-up of the use of tilt, it is understandable that participants want to be able to obtain feedback on the frequency, angle, and duration of tilt separately because this would let them target and address any problematic parameters. Finally, we should explore the possibility of including a dedicated space in the Web interface where users could note their daily and monthly experience with the use of tilt, similar to a logbook. The added value of this space should be evaluated carefully by considering the potential added burden on clinical follow-up and given the evidence of the proven use of this function [53-56].

### Limitations

This study has some limitations. First, only occupational therapists were recruited. This choice is explained by the fact that in Quebec (Canada), it is mainly occupational therapists who evaluate clients’ functional needs and who ensure training and follow-up of individuals who require mobility aids. It would be interesting to explore whether other categories of professionals would offer different insights. In addition, knowing that it could take several years before certain users consider their power wheelchair as an effective means to let them carry out significant activities [57], no attempts have been made to recruit new users of power wheelchairs, although clinicians who were interviewed identified these individuals as clients who could benefit from the intervention. In fact, the way new users of power wheelchairs experience the intervention may differ from that of more experienced users and thus influence the future development of the intervention. Finally, the conceptual framework proposed by Baumel et al [49] that underpinned the formative evaluation of our eHealth intervention puts more emphasis on users, whose targeted behavior is expected to change following the intervention, than on clinicians, who help users to attain the desired behavioral change. To fill this gap, during the analysis, we added emerging themes related to the needs identified by the clinicians. This conceptual framework could be enriched by integrating the perspectives of the staff who carry out the eHealth interventions.

### Conclusions

This study aimed to conduct a formative evaluation and to identify the functionalities needed by users of power wheelchairs and clinicians relative to a monitoring system designed to be installed on all models of power tilt-in-space wheelchairs. The results will orient the development of the prototype toward a more customizable monitoring system, with a more attractive and intuitive Web interface that favors communication between users and their clinicians. A formative evaluation involving a wider range of people such as new wheelchair users, users of manual tilt-in-space wheelchairs, children, the elderly, and family caregivers should be performed before evaluating the refined prototype in a real environment (eg, at home and in their daily life). Further research will also be necessary to evaluate if the intervention actually favors optimal use of tilt among power wheelchair users and improves the postprocurement monitoring offered by clinicians.

## Appendix

Multimedia Appendix 1 Tilt reminder system of the use of power tilt-in-space wheelchair.

## Abbreviations

BIT: Behavioral Intervention Technology
ehealth: electronic health
